# Excisional endometriosis surgery with hysterectomy and bilateral salpingo-oophorectomy versus excisional endometriosis surgery alone for pelvic pain associated with deep endometriosis

**DOI:** 10.52054/FVVO.15.1.055

**Published:** 2023-03-31

**Authors:** N Manobharath, J Lewin, M Hirsch, J Naftalin, A Vashisht, A Cutner, E Saridogan

**Affiliations:** University College London Medical School, United Kingdom; Elizabeth Garrett Anderson Institute for Women’s Health, University College London, United Kingdom; John Radcliffe Hospital, Oxford University Hospitals, Headley Way, Headington, Oxford OX3 9DU

**Keywords:** Endometriosis, rectovaginal endometriosis, excisional surgery, hysterectomy, pelvic pain

## Abstract

**Background:**

There is no agreed consensus on the optimal surgical treatment for pain associated with endometriosis.

**Objectives:**

To compare improvement in symptoms and quality-of-life in patients undergoing excisional endometriosis surgery (EES) versus EES with hysterectomy and bilateral salpingo-oophorectomy (EES-HBSO).

**Methods:**

This study evaluated patients undergoing EES and EES-HBSO at a single endometriosis centre between 2009 and 2019. Data was obtained from the British Society for Gynaecological Endoscopy database. Adenomyosis was assessed by blinded re-analysis of imaging and/or histology data.

**Main outcome measures:**

Pain scores (numeric rating scale 0-10) and quality-of-life scores (EQ-VAS) before and after EES and EES-HBSO.

**Results:**

We included 120 patients undergoing EES and 100 patients undergoing EES-HBSO. After controlling for baseline characteristics and the presence of adenomyosis, there was greater post-op improvement in non-cyclical pelvic pain amongst patients undergoing EES-HBSO compared to EES alone.The baseline pain scores had improved in the EES-HBSO cohort by 2.106/10 at 6 months (95%CI 0.469-3.742, p=0.012), 2.642/10 at 12 months (95%CI 0.871-4.413, p=0.004), and 2.548/10 at 24 months (95%CI 0.681-4.414, p=0.008), when compared to the EES group. Greater improvement amongst EES-HBSO patients was also seen for dyspareunia, non-cyclical dyschaezia and bladder pain. Patients undergoing EES-HBSO had greater improvement in EQ-VAS, although this was no longer statistically significant after controlling for adenomyosis.

**Conclusion:**

EES-HBSO appears to provide greater benefit than EES alone for symptoms including non-cyclical pelvic pain as well as for quality-of-life. Further research is required to determine which patients benefit the most from EES-HBSO, and whether removal of the ovaries, uterus or both is the key to this additional benefit in symptom control.

## Introduction

Pelvic pain associated with endometriosis impacts quality of life with limited data to support effective treatments for the condition (Becker et al., 2022). Amongst patients where medical treatments have been unsuccessful or unsuitable, laparoscopic excision of endometriosis is considered. Short- and medium-term follow-up data from specialist centres show significant improvement of pain and quality of life data (Byrne et al., 2018) However, endometriosis is considered a chronic condition and there is a significant risk of recurrence sometimes requiring repeat surgery (Ceccaroni et al., 2019).

Recommended surgical treatment of endometriosis usually involves laparoscopic or robotic excision of visible lesions, whilst preserving the uterus and ovaries. However, some patients opt to undergo more radical treatments including removal of the uterus, cervix, tubes, and ovaries. This induces a hypoestrogenic state and amenorrhoea, as well as removing adenomyosis which may be a concurrent cause of persistent pelvic pain (Dunselman et al., 2014; Ferrero et al., 2009).

Surgery for severe endometriosis is complex and associated with significant risks in the immediate operative period and longer term (Cea Soriano et al., 2017; Kondo et al., 2011), including a risk of visceral injury 4 times greater than surgery for other benign gynaecological conditions (Uccella et al., 2016). Radical surgery with premature removal of the ovaries is associated with increased long term cardiovascular and musculoskeletal morbidity (Parker et al., 2009). Conversely, disease recurrence, repeat surgery and persistent adenomyosis-related pain may limit the effectiveness of excisional endometriosis surgery (EES) with preservation of the uterus (Naftalin et al., 2016). Additionally, removal of the ovaries has the benefit of reducing the risk of ovarian cancer and may be associated with reduced reoperation rates for endometriosis- related pain (Asfour et al., 2022; Dunselman et al., 2014; Long et al., 2022).

The British Society for Gynaecological Endoscopy (BSGE) has established specialist endometriosis centres since 2007. This progressive approach to service delivery ensures centres meet pre-specified criteria to deliver specialist care to patients with severe endometriosis. These include a minimum number of procedures per annum per surgeon, a dedicated nurse specialist, named urological and colorectal colleagues for surgical support, and an annual exemplar video evaluation. This rigorous governance structure aims to ensure that standards of clinical and surgical care are maintained through regular evaluation and the collection of standardised data across the registered centres.

International guidance, based on expert opinion, recommends hysterectomy with or without removal of the ovaries for patients who no longer wish to conceive and who have failed to respond to more conservative treatments (Becker et al., 2022; Dunselman et al., 2014). However, there is limited evidence evaluating the effectiveness of excisional endometriosis surgery combined with hysterectomy and bilateral salpingo-oophorectomy (EES-HBSO) in comparison to conservative excisional surgery (EES).

The aim of this study is to evaluate the effectiveness of excisional endometriosis surgery with hysterectomy and removal of ovaries (EES- HBSO) for reducing symptoms and improving quality of life in comparison to EES alone.

## Methods

This study evaluated prospectively collected data on patients who underwent conservative excisional surgery and hysterectomy with bilateral salpingo-oophorectomy for pain symptoms associated with deep (Need to clarify what deep endo is) endometriosis. All patients were treated at University College London Hospital (UCLH), a BSGE-accredited tertiary endometriosis centre, by three accredited surgeons, using a laparoscopic approach.

We analysed prospectively-collected data originating from UCLH available on the BSGE database between January 2009 and June 2019 (unclear whether a retrospective data collection or as stated, prospective). Patients were divided into those who had undergone surgical excision of endometriosis (EES group) and those who had undergone surgical excision of endometriosis with removal of the uterus, tubes, cervix, and ovaries (EES-HBSO group). The decision on whether to perform EES or EES-HBSO was made in partnership with the patients on a case-by-case basis, based on their preferences, effectiveness of previous treatment, whether the patient felt that their family was complete, and other factors.

The patients who underwent EES-HBSO were offered hormone replacement therapy in the absence of specific contraindications. Data from a total of 100 consecutive patients undergoing EES-HBSO were analysed from the database, with 120 age-matched consecutive controls undergoing EES. We obtained guidance from NHS Research and Ethics. As there was no randomisation, no alteration in treatment offered or generalisable results, the study was considered to be a service evaluation and a separate ethics approval was not required. All patients had prospectively consented to the sharing of their BSGE data, and this study was approved by the BSGE Scientific Advisory Group (reference ID: BSGESAG2020-3).

### Data Collection

The BSGE database collects data on clinical symptoms at baseline (i.e., pre-operatively), and at 6-, 12-, and 24-months postoperatively. The symptoms include premenstrual pain, menstrual pain, non-cyclical pelvic pain, deep dyspareunia, cyclical dyschaezia, non-cyclical dyschaezia, lower back pain, bladder pain, pain during bladder voiding and quality of life. All patient-reported pain scores were assessed using a numeric rating scale from 0 (no pain) to 10 (most intense pain). Quality of life (QoL) scores were collected using the EuroQol visual analogue score (EQ- VAS)(Rabin and de Charro, 2009) from 0 (worst imaginable health) to 100 (best imaginable health).

Symptom and demographic data were obtained from the UCLH BSGE database. We compared only non-cyclical symptoms which are applicable to both EES and EES-HBSO groups, including non-cyclical pelvic pain, deep dyspareunia, non- cyclical dyschaezia, lower back pain, bladder pain, as well as overall quality-of-life as assessed by EQVAS score. We excluded patients with no preoperative pain scores, no postoperative follow-up and a previous history of hysterectomy. No patient was included twice in the study.

The presence of adenomyosis on ultrasound was assessed in a blinded manner by a consultant specialising in gynaecological ultrasound (JN), according to previously published criteria(Naftalin et al., 2016). Where a patient had undergone an MRI instead of an ultrasound, data from this investigation was included in determining presence of adenomyosis preoperatively, according to previously published criteria (Tellum et al., 2019). Presence of adenomyosis on histology reports of all patients that underwent EES -HBSO was also recorded.

### Statistical analysis

Baseline characteristics and symptom scores of patients undergoing hysterectomy and conservative surgery were compared using independent samples t–tests and Chi squared test of proportions. P-values of <0.05 were considered to be statistically significant throughout.

The study used a longitudinal multilevel modelling approach to investigate differences in improvement in symptom and EQVAS scores between patients undergoing EES with or without HBSO. Symptom and EQVAS scores were modelled with respect to time post-operation, using REML (restricted maximum likelihood) mixed-effect linear regression with a random intercept for each individual patient. Models were created in R using the lme function from the nmle package. Missing values were addressed with pair- wise deletion. The models were adjusted for age, smoking status, BMI, previous surgical treatment of endometriosis, diagnosis of adenomyosis and baseline symptom score.

## Results

Anonymised data of 737 patients were downloaded from the BSGE database. 22 duplicates were excluded from the study, with 38 patients excluded due to incomplete data: empty records (n=23) and missing hospital numbers (n=15). Other patients were excluded because they met one of the following exclusion criteria; no recorded preoperative pain scores (n=12), none of the follow-up pain scores were recorded (n=251) or previous hysterectomy (n=10). We identified 404 eligible patients of whom 296 had EES and 108 had EES with HBSO. After age-matching there were 120 patients in the EES group and 100 patients in the EES-HBSO group. Altogether, 220 patients were included in the primary analysis.

### Pre-operative symptom and demographic data

We evaluated preoperative demographics between the two groups. The mean BMI of patients undergoing EES-HBSO (mean 28; SD 4.89) was significantly higher than those in the EES group (mean 25; SD 5.03) (95%CI of difference 5.16 to 1.92, p<0.001). The mean age of patients who underwent EES and EES-HBSO were similar, 40 (SD 3.71) and 41 (SD 4.55) respectively. Current smokers represented 10% of the EES group and 8% in the EES-HBSO group (p = 0.607). History of previous surgery for endometriosis was common among both groups with 78% having had surgery in EES group and 88% in EES-HBSO group (p = 0.042). Adenomyosis was present in 44% of EES patients based on ultrasound and/or MRI findings whereas 57% of EES-HBSO patients had adenomyosis based on ultrasound, MRI and/or histology (Table I).

**Table I:** Baseline characteristics of patients who underwent EES and EES with HBSO. Group means shown with standard deviation in square brackets, and t-test or chi^2^ p-value.

		EES (n=120)	EES with HBSO (n=100)	p value
	Age^	40 [3.71]	41 [4.55]	0.90
	BMI^	25 [5.03]	28 [4.89]	<0.001
smoking – no. (%)	Current	12 (10)	8 (8)	
	Ex-smoker	17 (14)	25 (25)	
	Never	48 (40)	54 (54)	
	Unknown	43 (36)	13 (13)	
previous surgery – no. (%)	Surgical treatment for endometriosis	94 (78)	88 (88)	0.042
	One ovary removed	8 (7)	5 (5)	
	Both ovaries removed	1 (1)	0 (0)	
Adenomyosis No. (%)	Yes	53 (44)	57 (57)	<0.001
	No	27 (23)	38 (38)	
	Not available	40 (33)	5 (5)	

The mean preoperative symptom scores were significantly greater amongst those undergoing EES-HBSO compared to the EES group scores for premenstrual pain (EES 6.20/10 and EES-HBSO 7.00/10, p = 0.037); non-cyclical pelvic pain (EES 4.86/10 EES-HBSO 6.38/10, p < 0.001), deep dyspareunia (EES 4.32/10 and EES-HBSO 5.37/10, p = 0.036) and difficulty emptying bladder (EES 1.23/10 and EES-HBSO 2.18/10, p = 0.020). All other pain scores were similar between the two groups (Table II).

**Table II:** Baseline characteristics of patients who underwent EES and EES with HBSO. Group means shown with standard deviation in square brackets, 95% Confidence interval, and t-test p-value.

Symptom	Mean pre-operative pain score in EES group	Mean pre-operative pain score in EES-HBSO group	95% CI of difference	p value
Premenstrual pain	6.20 [2.85]	7.00 [2.71]	-1.55 to -0.05	0.037
Menstrual pain	8.11 [2.32]	8.33 [2.53]	-0.88 to 0.45	0.519
Non- cyclical pelvic pain	4.86 [3.01]	6.38 [2.80]	-2.30 to -0.75	<0.001
Deep Dyspareunia	4.32 [3.59]	5.37 [3.57]	-2.04 to -0.07	0.036
Cyclical dyschezia	5.99 [3.57]	6.40 [3.46]	-1.36 to 0.54	0.398
Non-cyclical dyschezia	3.77 [3.34]	4.14 [3.38]	-1.27 to 0.53	0.421
Lower back pain	6.05 [2.82]	6.47 [2.77]	-1.17 to 0.03	0.268
Bladder pain	2.23 [2.90]	2.68 [3.13]	-1.26 to 0.36	0.271
Difficulty emptying bladder	1.23 [2.38]	2.18 [3.35]	-1.74 to -0.15	0.020

The follow-up rates in EES and EES-HBSO were similar: 74% and 88% at 6 months, 65% and 63% at 12 months, and 46% and 49% at 24 months respectively.

### Symptom reduction after surgery

When mean pain scores were assessed at 6, 12, and 24 months postoperatively there was a reduction in pain at all time points compared to the preoperative pain scores irrespective of group, with the exception of difficulty emptying the bladder in the EES group where the symptom score increased at 12 months in comparison to the preoperative pain score.

Mixed effects linear regression was used to compare improvement in quality of life score (EQ-VAS) and pain scores between patients who underwent EES and patients who underwent EES-HBSO. The comparison was performed for pain scores at 6-, 12- and 24-months post-op and was adjusted for baseline characteristics of age, smoking, previous treatment of endometriosis and BMI, with and without adjustment for the effect of adenomyosis.

Non-cyclical pain improved significantly from baseline by 3.042/10 at 6 months post-op (95%CI 2.422-3.661, p<0.001), 2.515/10 at 12 months (95%CI 1.862-3.169, p<0.001) and 2.531 at 24 months (95%CI 1.841-3.223, p<0.001) overall. Compared to patients who underwent EES only, patients undergoing EES-HBSO had an additional improvement of 2.344/10 (95%CI 1.155-3.534, p<.001) at 6 months post-op, 2.624/10 at 12 months post-op (95% CI 1.372-3.877, p<.001) and 3.119 at 24 months post-op (95%CI 1.790-4.448, p<.001). After also controlling for the effect of adenomyosis, patients who had a hysterectomy still improved significantly more than patients who did not have a hysterectomy (Table III).

**Table III:** Table showing results of mixed-effects regression for non-cyclical pelvic pain scores adjusted for age, BMI, smoking and previous surgery. The effect of having a hysterectomy was estimated to result in an additional improvement in pain score by at least 2 points at all time-points compared to having excisional endometriosis surgery without hysterectomy, which remained significant after also controlling for the effect of a diagnosis of adenomyosis.

Time post follow-up	Additional symptom improvement amongst hysterectomy patients before and after adjusting for adenomyosis	95% CI	p-value
6 months	-2.469	-3.654 – -1.285	<0.001
12 months	-2.780	-4.026 – -1.534	<0.001
24 months	-3.286	-4.606 – -1.965	<0.001
After controlling for adenomyosis:			
6 months	-2.106	-3.742 – -0.469	0.012
12 months	-2.642	-4.413 – -0.871	0.004
24 months	-2.548	-4.414 – -0.681	0.008

For other symptoms, the symptom scores improved significantly more for EES-HBSO patients compared to EES for dyspareunia at 12 months by 2.306/10 (0.345-4.267, p=0.021), non-cyclical dyschaezia at 24 months by 1.857/10 (95%CI 0.138-3.575, p=0.034), and bladder pain at 24 months by 2.120/10 ( 95%CI 0.533-3.708, p=0.009). No other differences in symptoms were significant, and EES patients did not show greater improvement in symptoms than EES- HBSO patients at any time point for any symptom.

### Quality of life scores

Compared to baseline, overall mean EQ-VAS scores improved by 12.398/100 points at 6 months (95%CI 7.841-16.956 points, p<.001), 12.451/100 points at 12 months (95%CI 7.312-17.589, p<.001) and 10.369/100 points at 24 months (95%CI 5.558- 15.181, P<.001). Patients who underwent EES- HBSO had significantly greater improvement in EQVAS score at 24 months compared to patients who underwent EES only, by 10.285/100 points (95%CI 0.038-20.532 p=.049) (Table IV, Figure 2). However, after controlling for adenomyosis, the difference in EQVAS score improvement between the EES and EES with HBSO groups was no longer significant.

**Table IV:** Table showing results of mixed-effects regression for EQVAS scores adjusted for age, BMI, smoking and previous surgery. The effect of having a hysterectomy was estimated to result in an additional 10 .3 points in EQVAS score at 24 months post-op compared to having excisional endometriosis surgery without hysterectomy, although this effect was no longer significant after also controlling for the effect of a prior diagnosis of adenomyosis.

Time post follow-up	Additional symptom improvement amongst hysterectomy patients before and after adjusting for adenomyosis	95% CI	p-value
6 months	-1.619	-10.752 – 7.514	0.727
12 months	7.091	-2.503 – 16.685	0.147
24 months	10.285	0.038 – 20.532	0.049
After controlling for adenomyosis:			
6 months	-3.384	-15.198 – 8.429	0.572
12 months	4.791	-7.998 – 17.580	0.460
24 months	7.347	-6.440 – 21.134	0.294

**Figure 1:**
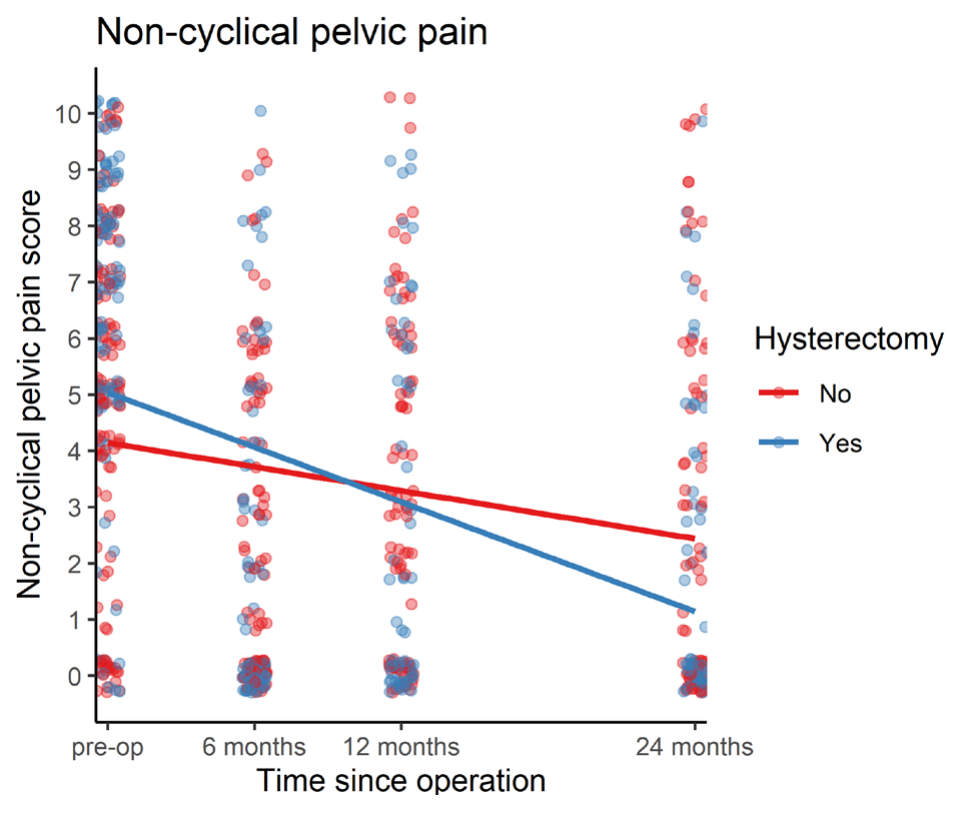
Overall improvement in non-cyclical pelvic pain scores in patients who had excisional endometriosis surgery with and without a hysterectomy.

**Figure 2:**
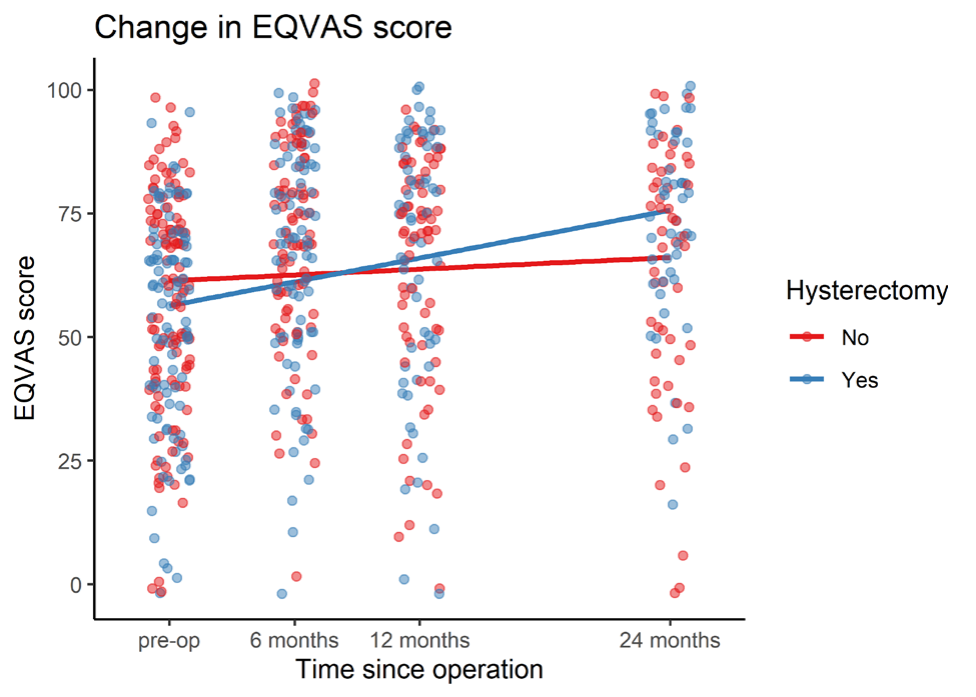
Overall improvement in EQVAS quality of life scores in patients who had excisional endometriosis surgery with and without a hysterectomy.

## Discussion

### Key Findings

Our study demonstrates that there may be additional benefit of EES-HBSO over EES alone for the surgical treatment of pain associated with endometriosis. Patients who underwent EES-HBSO saw greater improvement in non-cyclical pain at all follow-up time-points compared to those patients undergoing EES alone. This effect remained significant even after controlling for the presence of adenomyosis. The benefit of EES-HBSO was also seen in symptom scores for deep dyspareunia, bladder pain and non-cyclical dyschaezia. EES- HBSO patients showed greater improvement in quality-of-life score compared to EES patients after controlling for baseline characteristics but not after controlling for the presence of adenomyosis, indicating that this additional improvement was mostly seen in patients with adenomyosis.

### Strengths and Limitations

This is the first study to compare prospectively collected long-term pain and quality of life outcomes amongst patients with deep endometriosis undergoing EES versus EES-HBSO. Previous studies have compared re-operation rates but not improvement in symptoms (Bougie et al., 2021b; Shakiba et al., 2008), while another study observed symptom improvement amongst hysterectomy patients, but with no comparator group (Sandström et al., 2020). With a sample size of 220 patients, we were able to demonstrate statistically significant additional benefits of EES-HBSO over EES alone. The study was conducted in a single BSGE endometriosis centre where all patients were treated by a small number of accredited surgeons, ensuring consistency in surgical care. Prior history of surgery was common and may reflect the specialist nature of this individual endometriosis centre attracting referrals of complex cases from other centres, however findings remained consistent after controlling for previous surgery. All patient-reported outcomes were prospectively collected, reducing interpreter bias and recall bias.

As an observational non-randomised study, selection bias inevitably affected results, and accordingly worse baseline pre-operative symptoms and quality of life were found amongst patients who underwent EES-HBSO compared with those who underwent EES. A limitation of our study includes lack of data on the duration of symptoms experienced by the patients, which may have differed between groups, as well as the stage of endometriosis. Patients with worse or more long-standing chronic pain would be expected to show less overall improvement than those with less severe symptoms (Sullivan et al., 2008), however in spite of this greater improvement in symptoms and quality-of-life was found amongst patients undergoing EES-HBSO despite worse symptoms at baseline.

All patients in our study who had a hysterectomy also underwent bilateral salpingo-oophorectomy, therefore it is not clear from the data whether it is the removal of uterus or ovaries which was providing the key benefit. Further work comparing these two treatment modalities is required to determine whether symptom improvement can be attributed to surgical menopause through oophorectomy or removal of adenomyosis by hysterectomy.

This data is not definitive, and a starter point for further work to confirm whether EES-HBSO may provide additional benefit over EES alone for the control of symptoms and for which patient group. Hysterectomy results in irreversible loss of fertility, so the importance of further research to inform this choice cannot be stressed enough. Ideally the benefits of hysterectomy over EES alone would be studied in a randomised controlled trial, however this may not be ethically feasible, and may still suffer from selection bias at the enrolment and follow-up stages. Additionally, although greater improvement in pain symptoms were shown amongst EES-HBSO patients even after controlling for the presence of adenomyosis, this must be treated with caution as data on adenomyosis was missing from 33% of those undergoing EES alone. While both EES and EES- HBSO patients in our cohort may have undergone pre-operative imaging, histological examination of the uterus for adenomyosis was only possible for patients undergoing EES-HBSO. As the sensitivity of TVUS and MRI for adenomyosis is only 81% and 71% respectively (Liu et al., 2021), this may have resulted in a higher rate of diagnosis of adenomyosis in EES-HBSO patients. Underdiagnosed adenomyosis may have played a role in the lower rates of symptom resolution seen in the EES group, and particularly if hormonal suppressive therapy was not used post-operatively.

Follow-up was not complete and fell from 81% at 6 months, to 64% at 12 months and finally to 48% at 24 months. It was decided to not contact patients in order to obtain symptom questionnaires, as this may introduce recall bias, so loss to follow-up remains a limitation of this study. Maintaining long-term follow-up is a challenge, particularly in this relatively young and mobile patient group. However, the follow-up data in our cohort was considerably more complete than the average follow-up rates of 50%, 38% and 27% at 6-, 12- and 24-months post-op respectively for the BSGE database overall (Byrne et al., 2018).

### Interpretation

Our findings are consistent with other studies which confirm that hysterectomy is a valuable treatment option for patients with pain symptoms associated with endometriosis (Martin, 2006; Sandström et al., 2020; Shakiba et al., 2008). A recent long- term follow-up population study highlighted that less than 2% of patients undergoing hysterectomy for endometriosis went on to require additional surgery, while approximately 20% of patients having conservative EES underwent additional surgery within 5 years of index procedure (Bougie et al., 2021a).

These studies and ours demonstrate the utility of hysterectomy with bilateral salpingo- oophorectomy in effectively treating symptoms, improving quality of life while reducing the need for repeat surgery. This treatment strategy mirrors internationally recommended medical approaches of inducing a hypoestrogenic state (Dunselman et al., 2014). While these are temporary and reversible, hysterectomy prevents future fertility and will not be suitable for many patients who wish to conceive. Furthermore, removal of the ovaries induces menopause, which presents several problems, including bone mineral density loss and adverse cardiovascular effects (Cusimano et al., 2021; Faubion et al., 2015). Many of these issues can be mitigated with hormone replacement therapy, for which evidence demonstrates a number of benefits including prevention of osteoporotic fractures (Kurabayashi et al., 1998; Marjoribanks et al., 2017) and cardiovascular events (Schierbeck et al., 2012). Although recurrence of endometriosis after hysterectomy due to hormone replacement has been reported, this is rare and affects up to 5% of such patients (Hickman et al., 1998; Matorras et al., 2002). When comparing these two approaches studies have previously found an 8.1-times increased risk of reoperation following preservation of ovaries (Namnoum et al., 1995). This is again reflected in recent studies highlighting a significantly lower recurrence rate amongst patients undergoing oophorectomy compared to hysterectomy with ovarian preservation (Fedele et al., 2005; Shakiba et al., 2008).

It is unclear whether patients are benefitting from removal of the uterus, the ovaries or both. Historic comparative data indicates that hysterectomy with or without ovarian preservation reduces reoperation rates (Shakiba et al., 2008). However, whether the improvement in pain after hysterectomy for endometriosis is influenced by the preservation or removal of ovaries remains uncertain (Sandström et al., 2020). In our studied cohort, all hysterectomy patients had removal of their ovaries after careful counselling regarding risks and benefits and discussion of alternatives, which is the department’s unified approach in line with the previous ESHRE (acronym) guidance (Dunselman et al., 2014). Further work, ideally in the form of randomised-controlled trials, is necessary to determine whether removal of the ovaries, uterus or both is the optimal strategy for symptom control in patients with severe endometriosis, as well as the role of GnRH analogues post-surgery. Additionally, study of a larger cohort of patients would allow detailed sub- group analysis to identify factors which influence patient response to hysterectomy compared to EES alone.

Another cause for consideration is the added operative complexity that hysterectomy entails, especially as complication rates are higher from hysterectomy for endometriosis compared to hysterectomy for other benign indications (Brunes et al., 2022; Casarin et al., 2021; Stewart et al., 2022). In a large retrospective legal claims study comparing hysterectomy with laparoscopic excision of endometriosis, hysterectomy was associated with a higher risk of immediate post-operative infection, pulmonary embolus, and fistula formation, although these complications were rare (Surrey et al., 2017). A population-based cohort study also found that patients undergoing hysterectomy for endometriosis had nearly twice the length of stay and 30-day complication rate than those undergoing conservative surgery (Bougie et al., 2021a). Assessment of this additional surgical risk should nevertheless take into account the reduced need for reoperation after hysterectomy, which may negate the overall lifetime risk of suffering such complications when multiple operations are taken into account. In addition, hysterectomy may enable the surgeon to carry out a more complete excision of endometriosis due to providing better excess to parametria and rectovaginal space. It is possible that this advantage may be the reason for lower recurrence and re-operation rates following hysterectomy.

Previous studies have also suggested the immediate risk of bladder dysfunction increases proportionately with complexity of endometriosis surgery (Kovoor et al., 2010; Li et al., 2014). This may reflect greater surgical complexity associated with hysterectomy resulting in injury to the pelvic nerves orchestrating bladder function (Vashisht et al., 2009). We note from our data a trend towards increased post-operative bladder pain and voiding difficulties in patients undergoing EES-HBSO, however differences were small and not statistically significant, which provides reassurance for patients considering EES-HBSO as an option.

## Conclusions

This study presents the first and largest comparison between excision of endometriosis alone versus with hysterectomy for the treatment of deep endometriosis associated pain. Patients who underwent EES-HBSO achieved greater improvements in their symptoms and quality-of- life scores than patients undergoing solely excision of endometriosis. The added benefit in symptom control must be weighed against the complication rate and reproductive and hormonal side-effects on an individual basis. This study will help inform patients about their available choices and enable an open discussion about expectations after EES and EES-HBSO.
